# Prof. T. S. Powell’s Lecture to the Medical Class at the Southern Medical College, Atlanta, Ga., Oct. 1883

**Published:** 1883-12-20

**Authors:** 


### Southern Medical Record:

EDITORS:

T. S. POWELL, M.D. W. T. GOLDSMITH, M.D. R. C. WORD, M.D.

*R. C. WORD, M.D., Managing Editor.*

☞ All Communications and Letters on Business connected with the RECORD must be addressed to the Managing Editor.

VOL. XIII. ATLANTA, GA., DECEMBER 20, 1883. No. 12.

### ORIGINAL AND SELECTED ARTICLES.

### PROF. T. S. POWELL'S LECTURE TO THE MEDICAL CLASS AT THE SOUTHERN MEDICAL COLLEGE, ATLANTA, GEORGIA, OCTOBER, 1883.

GENTLEMEN OF THE CLASS:

As the word farewell is considered to be one of the saddest of earth, so the salutation, "Welcome" thrills the heart of men with a glad and comfortable sense of greeting.

The warm grasp of the hand is more eloquent than words; the glad light in the eyes of a friend and the cordial smile on his lips, at your approach, are expressions for which there is no need of an outburst of language.

And yet, gentlemen, I say to you, "Welcome, most welcome!" to the Southern Medical College; and may you draw from its bosom all the nourishment, strength and vigor your Medical Alma Mata should give to her offspring.

I am truly glad to see before me the faces of so many students who were with us at the last session; both glad and proud, because their presence here again attests the fact that they are pleased with the Southern Medical College, and are faithful in their patronage of this institution. So, to them we give the greeting due to tried friends, while we also extend the most cordial welcome to the many new matriculates that are with us to-day. I trust that our position towards each other, as instructor and pupils, may be of mutual pleasure and profit, without any discordant incidents to interrupt the harmony of the relation.

While I shall endeavor to give you faithful instruction in my department of medical science, I ask and believe that in return you will do justice to yourselves and the institution, by patient and persevering application to the study of your chosen profession. To it you owe supreme devotion; so do not allow the pleasures of the world to entice you from the performance of your duties and obligations, but let your character as students and as gentlemen be without reproach in the eyes of your class-mates, your instructors and the public.

No doubt you all have loved ones at home who will watch your course while students in this institution, with that anxious interest which only can be felt by those who are nearer and dearer to you than any friends the world can give. Respect, then, their hopes, and their high anticipations for you in your enforced absence.

Have a reverence for the church your mother loves, and the God whom she serves; and after a pleasant and profitable season among us, may you return to your homes in health, happiness and with good report in all things.

You have come, gentlemen, to prosecute your medical studies in a city whose marvelous growth, public spirit, energy and enterprise challenge the admiration of men throughout our sunny land. With an ever-aspiring spirit of emulation, Atlanta would have no competitor to surpass her in the highest achievements of progress and excellence; so, while with us, gentlemen, there will be around you so many examples of a noble ambition, your mind and purposes should become instinct with their inspiration until you feel that if you, too, do not have these ardent aspirations in your especial life-work, you will be laggards in the race, and fail to reach the goal of successful emulation.

You are in a city that is rapidly advancing to its inevitable destiny as the cosmopolitan centre of the State and of the South. All around you are seen striking evidences of the vim and enterprise that led to success and great achievements.

The air about us is vocal with the inspiring notes of a vigorous and ambitious spirit of progress.

The scores of panting, ever-restless locomotives upon our seven railroads, leading into the very heart of the city; the whirr of machinery in our numerous manufactories, and the numerous branches of trade and commerce in our midst, tell with trumpet tongue to the world that the Gate City of the South, though young in years, is athletic in strength, and fast becoming gigantic in statue.

Its splendid public and private schools and numerous literary societies; its magnificent public library; its thirty or forty successful newspapers and periodicals, and its liberal, enthusiastic patronage of music and all the fine arts, bespeak the intelligence and broad culture manifest in that refined and elevated tone which distinguishes our social circles.

Ours is also a city of churches, well attended and generously supported; and Atlanta has the reputation of being the most moral city of its size on the continent. In addition to all these, we might speak of our broad and handsome streets, thronged with benevolent, generous and warm-hearted men and women, of our magnificent public buildings, and the many beautiful residences of the cultured and wealthy; then conceive all these admirable features concentrated in a city literallv set upon more than "seven hills," with a climate mild in winter, temperate in summer, and yet so pure and bracing, and so free from malaria and the prevalence of epidemics, it is emphatically true that the locality of Atlanta makes it the healthiest city, perhaps, in the world; most desirable in all respects, and especially so as a commercial, social and educational centre.

Good men of all classes and conditions are, therefore, encouraged to come to Atlanta. Men of wealth and refinement may here increase their wealth, while they may find enjoyment in our high educational and social advantages, and in the almost unparalleled salubrity of her climate.

Professional gentlemen, scholars and scientists will here find congenial companions in the circles of learning, and ample facilities for prosecuting their studies.

The industrious man, the artisan, the mechanic and the laborer may here find a ready welcome, with abundant avenues open to advancement and success in any and every field of enterprise and of usefulness. To laboring men—more, perhaps, than to any other class—the mildness and healthfulness of the climate rendering it practicable to work at all seasons and throughout the entire year, Atlanta possesses marked and Superior advantages.

What more can be desired of a community and of a city than what we have truthfully stated in respect to Atlanta?

The SOUTHERN MEDICAL COLLEGE, an Institution which its founders have proposed from the beginning to make a shining light to the profession of the South, and the exponent of that fuller knowledge and higher medical education which the progress of the age, the exigencies of the times, and the educated popular mind demand of both instructors and students in the science of medicine, has been wisely and happily located in this city.

It is designed to have this institution second to none as a medical school, and each year we are making rapid strides toward that goal of our hopes.

Our well-constructed and commodious college building, with its central position in the city, is convenient to all of onr street railroads, hotels and many boarding houses, yet is sufficiently private for all purposes. Our large lecture-rooms, well-ventilated and lighted with gas, have all the requisite modern conveniences, and our chemical laboratory is complete for instruction in that important department. The hall of the museum is stored with the necessary anatomical preparations, curiosities, relics and specimens; and our dissecting-room, elevated, well supplied with water, and brilliantly lighted, is one of the best, if not the best in the union.

The Central Ivy Street Hospital, which is under the medical charge of the Faculty, affords exceptional opportunities for clinical instruction to the Class. To any intelligent mind, informed upon the subject, it is needless to speak of the great importance and advantages of this acquisition to the Southern Medical College, since practitioners and students will at once appreciate the importance of this method of instruction to those who are seeking a really thorough medical school.

The Hospital adjoins the college lot, making it easy of access, and enabling the Faculty to have cases brought from the hospital to the college and exhibited to the class. In this way the Faculty are enabled, during the whole session, to utilize every case to the best interests of the patients and of those receiving instruction at their hands.

With all these facilities, no one should be surprised at the rapid progress the College has made since it was opened four years ago; and we feel most grateful for the confidence and co-operation of the Profession, and the high estimate in which they hold the Institution. To merit that confidence and meet the increasing demands upon us, the Trustees and Faculty will spare no effort nor expense to enlarge our facilities and to make THE SOUTHERN MEDICAL COLLEGE an honor to the South, and to the profession at large.

The demand for teachers of first-class character and attainments in their respective departments of instruction, is one of the gratifying signs of the times. It shows that the popular mind is aroused upon the subject, and calls for instructors who are teachers in the truest sense of the word—who have not only high scholastic acquirements in their especial work, but who possess the art of imparting their knowledge to the pupil in a clear, concise and practical manner, so there will be no difficulty in comprehending and retaining the instruction given. Knowledge thus acquired by the student will enable him to give his own demonstrations and utilize them to the best purposes by combining theory and practice in his work.

I will confess that but comparatively few instructors are endowed by nature with this happy art, but it can be cultivated and acquired to a great extent, and should be regarded as an indispensable qualification in a teacher.

A man may make a brilliant oration upon medical science, but it will prove of little value to the student of medicine unless another man comes after him and demonstrates what the former has merely expressed in ornate language, though replete it may be with thoughts of significance and beauty.

Another index of the exalted views to which popular thought is more and more directed, is the demand for *morality* in the instructor, and consequently a good moral influence upon the student. It should inspire the souls of men to note this reaching up from a low, earth-bound plane of action, to a higher and purer atmosphere in which to do man's work, and thus exalt the standard of the profession in its moral not less than in its scientific aspects.

He who will read these indications of the times, will see that the tendency is to educate the mind up to a love of order, beauty and symmetry, and to the highest excellence with all those pleasing features which are attractive to the true artist in any work, however practical that work may be. Hence the public, to a greater extent than ever before, is becoming disgusted with the coarseness and the immorality, the foulness and the hideous deformity which were too often apparent in the older methods of teaching.

With the exception of a minister of the gospel, no man should have a higher moral character than the teacher in any branch of instruction.

Any man who has not wholly lost his self-respect, is shocked to hear his son utter a profane oath, though he himself stains his lips and his soul with the sin. As a teacher's influence, in the close contact with his pupil, will go far towards elevating or bringing low that pupil's moral standard, a father, though a bad man himself, never prefers the teacher who will set his boy the example of drunkenness, profanity, gambling and other vicious habits.

Besides, no man's character can be rounded, complete and symmetrical unless he is of strict moral integrity, a true gentleman. Then he may be called a man, indeed, in all the grand and noble significance of the word; and he stands out before the world in bold relief, a beautifully carved figure of marble-white purity against the dark background of sin and vice.

Gentlemen of the class, allow me to urge this upon you—whether in private life, or in your profession: Try to have your character moulded into this statue of moral strength, and beautiful, symmetrical proportions. Keep this always in your mind, that every man is no better and no greater than he wishes to be; hence his character and his attainments are no lower or higher than are his aspirations and his efforts. *Strive to excel;* for even should you fail in some of your endeavors, the very act of making the effort will strengthen, purify and exalt your character as a man and a professional gentleman. But no good work is ever lost, and if not in the near future, sometime in the coming years you will gather the fruits of your labor, and have your reward.

In the profession you have chosen, you will bear a relation toward woman, as counsellor and preserver, which will reveal to you much of her mental and emotional nature, as well as her physical ills; and when you approach this mysterious and sacred arcana, overshadowed by the very presence of the Deity you should come with a pure heart, clean hands and a reverent spirit. Then you will be fitted to become truly her friend, as well as adviser, as every man should be who ministers to the dual combination of woman's mental and physical idiosyncrasies.

I am glad that medical scientists are giving more thought and investigation to this subject. When we learn to make woman vigorous, healthy and buoyant in both mind and body, and, consequently, happy and useful in fulfilling her true destiny, then we become the benefactors, without a peer, of all humanity. I am certain that a great deal of woman's physical weakness and unhappiness is caused by men not understanding her needs and her nature.

At her worst, she is better than the most of men, and men should make it a business to study the wonderful phases of her womanhood, and ascertain how it is best to promote her health, happiness and usefulness in the important relation she sustains toward man.

It has been often said that good women make good men, but I feel assured that every wicked woman had some bad man for an example or a leader. You may cite the case of our first Mother, and I will answer that we all know the Devil, in the guile of a serpent, was of the *male* persuasion.

I have known a woman who, for nearly forty years, tried to persuade her husband to become a good man, and he rejected her petitions every time. But when a man asks his wife, mother or sister to join him in living a better life, in nine cases out of ten she will follow his lead promptly and gladly, and with all her heart and soul. So, woman's nature, whatever its faults may be, is better and higher than that of man, and he can never be her equal until his life is assimilated to those higher and purer instincts which characterize the gentler sex.

Whether as an angel of compassion and mercy in the hovels of the poor, as a shining example of womanly virtues and accomplishments in social circles, as the loved and honored queen of home in the character of tender mother, devoted sister or faithful wife, woman is the promoter of the truest civilization, the conservator of morals and the vestal of her country's altars: in a word, she is the last wonderful *thought* of God, crystalized into a form of angelic loveliness, and sent to cheer and to bless, to guide and sustain, electrify and lighten the sin-darkened world of mankind.

All honor, then, to self-sacrificing and devoted woman. Young gentlemen, forget not, I beseech you, in the hard and fearful battles of life, the eternal obligations you owe to her who gave you birth, nurtured you in youth, and strewed bright flowers of love in your pathway to manhood: who stood beside your couch in sickness, and pointed your soul to the bright stars of Paradise that cluster around the Throne of God! Her smile shed a halo of glory around the days of your childhood, and her tears are the brightest offerings at the gates of the Celestial City; while her prayers in your behalf ever ascend, like incense, to Heaven. Remember, to have had a good mother is the stepping stone to honorable success in this life and an immortality of bliss in the life to come.

---

**Phosphate of Codein.**—The *Wiener Med. Blatter* for August 16th contains the mention of a new drug, the phosphate of codein, which has been prepared by Merck, of Darmstadt, under the direction of Professor Hegar, of Freiburg. It is intended for hypodermic injection, for which neither the sulphate nor the chlorate are suitable, being nearly insoluble in water. The new salt is soluble in four parts of water, and contains seventy per cent. of codein. It crystallizes in four-sided columns, and is similar to morphia in action, with the advantage of having less tendency to excite toxic symptoms. It is particularly suitable for sensitive patients.—*N. Y. Med. Rec.*

